# Unveiling the Hidden Burden: Exploring the Psychological Impact of Gynecological Cancers and Predictive Modeling of Depression in Southwest China

**DOI:** 10.1155/2024/6512073

**Published:** 2024-08-02

**Authors:** Xingyu Sun, Shiqi Jiang, Beibei Jiao, Peijuan Wang, Qiong Wang, Lijuan He, Chengliang Yin, Ling Liu, Shaohua Wang

**Affiliations:** ^1^Department of Gynecology, The Affiliated Traditional Chinese Medicine Hospital, Southwest Medical University, Luzhou, Sichuan 646000, China; ^2^Department of Anesthesiology, The First Hospital of China Medical University, No.155 Nanjing Road, Heping Area, Shenyang, Liaoning Province, China; ^3^Affiliated Hospital of Integrated Traditional Chinese and Western Medicine, Nanjing University of Chinese Medicine, Nanjing 210028, China; ^4^Tonglu Hospital of Traditional Chinese Medicine, Tonglu 311500, China; ^5^Department of Critical Care Medicine, Southern University of Science and Technology Yantian Hospital, Shenzhen, China; ^6^Department of Health Management Center, The Affiliated Hospital, Southwest Medical University, Luzhou, Sichuan, China; ^7^Faculty of Medicine, Macau University of Science and Technology, Macau 999078, China; ^8^Department of Reproductive Medicine Center, The Affiliated Hospital, Southwest Medical University, 25 Taiping Street, Luzhou, China; ^9^Department of Pathology, The Affiliated Hospital, Southwest Medical University, 25 Taiping Street, Luzhou, China

## Abstract

**Objective:**

To explore the psychological impact of gynecological cancers on middle-aged women in Southwest China and identify the risk factors for moderate to severe depressive symptoms.

**Methods:**

This cross-sectional study included 500 patients from Southwest China, divided into two groups: depression (*n* = 220) and no depression (*n* = 280). Data on demographics, clinical characteristics, and socioeconomic factors were collected. We developed a logistic regression model to predict depressive symptoms and assessed its accuracy using the area under the receiver operating characteristic curve (AUC).

**Results:**

The study cohort consisted of 500 middle-aged and young female cancer patients with a median age of 44 years. Significant predictors of depressive symptoms included younger age, higher economic stress levels, and out-of-pocket medical expenses. A comparative analysis showed that 220 patients exhibited depression symptoms, with these patients being generally younger (median age 41 years) compared to those without depression (median age 47 years, *p* < 0.001). Economic stress was consistently higher in the depression group across all cancer types. Patients with ovarian cancer had a reduced risk of depression compared to those with cervical cancer. The predictive model demonstrated high accuracy in identifying depression risk, with an AUC of 0.888. Internal validation yielded an average AUC of 0.885, and external validation produced an AUC of 0.872, underscoring the model's robustness and reliability. These findings emphasize the complex interplay of demographic, socioeconomic, and clinical factors in the psychological well-being of gynecological cancer patients, highlighting the need for tailored psychological and financial support interventions.

**Conclusion:**

Gynecological cancer patients in Southwest China experience significant psychological challenges, particularly younger women and those facing economic stress. Our predictive model can aid in early identification of those at risk for depression, emphasizing the importance of holistic care. Interventions should focus on both psychological and financial support to improve patient outcomes.

## 1. Introduction

Gynecological cancers, encompassing malignancies of the ovary, uterus, cervix, vulva, and vagina, are among the most prevalent and consequential health challenges faced by women globally [1]. These cancers not only bring forth a physical burden but also usher in a myriad of psychological implications, among which depression stands paramount [2]. Depression, characterized by persistent sadness, a lack of interest in activities, and a variety of emotional and physical problems, has consistently been shown to negatively impact the quality of life, treatment adherence, and even survival rates among cancer patients [3, 4].

In the context of gynecological cancers, the intimate nature of the disease, coupled with its potential implications on fertility, sexual function, and body image, can particularly heighten the risk of depressive symptoms [5, 6]. Moreover, the age at which these cancers strike can also play a pivotal role in determining the psychological outcome. Younger and middle-aged women, often in the prime of their lives juggling multiple responsibilities, can find the diagnosis especially overwhelming [7].

While several studies have explored the prevalence and determinants of depression among cancer patients [8], including large-scale studies that compare cancer subtypes and related depression [9, 10], there is still a need for research specifically focusing on gynecological cancer patients in the context of middle-aged women in China [9, 10]. Most existing research has predominantly focused on Western populations, leaving the experiences and challenges of Asian women, especially those from China, underrepresented [11].

In light of these considerations, our study aims to identify the risk factors associated with moderate to severe depressive symptoms among middle-aged women diagnosed with gynecological cancers in China. By establishing a robust predictive model, we intend to pave the way for early identification of high-risk individuals, facilitating timely interventions and thereby improving the overall quality of life and prognosis for these patients.

## 2. Materials and Methods

### 2.1. Study Design and Participants

This cross-sectional observational study was conducted at The Affiliated Hospital of Southwest Medical University from January to December 2021. We recruited a total of 500 middle-aged women diagnosed with gynecological cancers. The inclusion criteria were female patients aged 18–60 years, diagnosed with gynecological cancer, admitted during the study period, and willing to provide informed consent. Exclusion criteria included concurrent malignancies, cognitive impairments or psychiatric disorders that impeded valid responses, and recent psychiatric treatment or antidepressant use.

### 2.2. Data Collection

Data were collected through structured interviews using a standardized questionnaire. The questionnaire covered the following variables:

- Demographics: age, education level, marital status, and number of children.
- Clinical variables: type of cancer diagnosis, cancer stage, and presence of chronic diseases.
- Socioeconomic and psychosocial variables: primary caregiver, work status, method of medical payment, and economic stress level.

### 2.3. Depression Assessment

Depression status was assessed using the PROMIS depression short form. Patients were categorized into two groups based on their *T*-scores: depression (*n* = 220) and no depression (*n* = 280). A *T*-score threshold of 60 was employed, indicating moderate to severe depressive symptoms. This threshold is based on the PROMIS guidelines, where a *T*-score of 60 or above represents a level of depressive symptoms that is clinically significant and one standard deviation above the mean of the general population. By employing the *T*-score threshold of 60, we ensure that our classification of depression is consistent with established research practices, thereby reinforcing the reliability and comparability of our findings with existing literature. This approach allows for early identification and intervention for patients at risk of significant depressive symptoms, ultimately contributing to better patient outcomes and quality of life.

### 2.4. Statistical Analysis

All data were analyzed using R statistical software (version 4.1.2, http://www.r-project.org). Descriptive statistics, including medians and interquartile ranges (IQR) for continuous variables and frequencies and percentages for categorical variables, were used to summarize the characteristics of the study population. We conducted univariate and multivariate logistic regression analyses to identify factors associated with depression symptoms in middle-aged and young female cancer patients. In the multivariate analysis, we adjusted for the following potential confounders: age, marital status, education level, number of children, primary caregiver, work status, medical payment method, economic stress, cancer diagnosis, cancer stage, and the presence of chronic disease. These confounders were selected based on their potential influence on the psychological well-being of cancer patients and their relevance to the study population. The inclusion of these variables aimed to control for their impact and ensure that the associations identified were independent of these confounding factors. The predictive performance of the logistic regression model was evaluated using the area under the receiver operating characteristic (ROC) curve (AUC), which measures the model's ability to discriminate between patients with and without depressive symptoms. Internal validation was performed using 10-fold cross-validation, where the dataset was randomly divided into 10 subsets, with the model being trained on nine subsets and tested on the remaining subset, repeated 10 times to obtain an average AUC and its standard deviation. External validation was conducted using an independent dataset of 200 gynecological cancer patients from a different time period and location, and the model's performance on this external dataset was assessed by calculating the AUC.

### 2.5. Model Validation

To ensure the robustness and generalizability of the predictive model, both internal and external validation were conducted:

- Internal validation: We performed 10-fold cross-validation on the original dataset. The data was divided into 10 subsets, with the model being trained on nine subsets and tested on the remaining subset in each iteration. The AUC for each fold was calculated, and the mean AUC, 95% confidence interval, and standard deviation were computed to assess the model's stability and reliability.
- External validation: An independent dataset comprising 200 additional gynecological cancer patients, collected from a different time period and location within Southwest China, was used for external validation. The model's performance on this dataset was evaluated using the AUC. The standard deviation was calculated to provide a measure of variability and to compute the 95% confidence interval.

## 3. Results

### 3.1. Comparative Analysis of Demographic and Clinical Characteristics between Patients with and without Depression Symptoms

The demographic and clinical characteristics of 500 middle-aged and young female cancer patients are presented in Table 1, comparing those without depression symptoms (*n* = 280) and those with depression symptoms (*n* = 220). The median age of participants was significantly different between the groups, with the depression group being younger (median age 41 years, IQR: 29–52) compared to the no depression group (median age 47 years, IQR: 33–58; *p* < 0.001). Education levels varied among the participants, but no significant difference was observed between the groups (*p*=0.388). Marital status, number of children, primary caregiver, and work status also did not show significant differences between the groups. However, significant differences were observed in the method of medical payment and economic stress levels. A higher proportion of patients in the depression group paid out-of-pocket for medical expenses (47.3% vs. 32.1%), and the median economic stress level was higher in the depression group (median of 4, IQR: 3–5) compared to the no depression group (median of 3, IQR: 2–4; both *p* < 0.001). Additionally, 77.3% of patients in the depression group reported having a chronic disease, compared to 67.9% in the no depression group (*p* < 0.001). Regarding cancer diagnosis, there were no significant differences in the distribution of different types of gynecological cancers between the groups (*p*=0.203). Similarly, cancer staging did not show significant variation between the groups (*p*=0.434).

### 3.2. Stratified Analysis of Demographic and Clinical Characteristics

Tables S1 and S2 present the demographic and clinical characteristics of middle-aged and young female cancer patients, stratified by type of gynecological cancer, and a comparative analysis of patients with and without depression symptoms for each type of gynecological cancer. The data show that while the overall demographic and clinical characteristics were relatively consistent across different cancer types, there were some notable variations. For instance, ovarian cancer patients had a slightly younger median age, and a higher proportion of vulvar cancer patients paid out-of-pocket for medical expenses. In the comparative analysis, significant differences emerged between patients with and without depression symptoms. Depressed patients were generally younger across all cancer types, with cervical cancer patients showing a median age of 42 years in the depression group compared to 48 years in the nondepression group, and similar trends were observed for uterine, ovarian, and vulvar cancer patients. Economic stress levels were consistently higher in the depression group across all cancer types. For example, the depression group of cervical cancer patients had a higher economic stress median (4) compared to the nondepression group (3). Additionally, the method of medical payment revealed significant differences, with a higher percentage of depressed patients paying out-of-pocket across all cancer types. For instance, 33.3% of depressed cervical cancer patients paid out-of-pocket compared to 25.6% in the nondepression group, and similar patterns were seen in uterine, ovarian, and vulvar cancer patients. These findings underscore the importance of considering the specific type of gynecological cancer when examining risk factors for depression. Younger age and economic stress were consistently associated with higher rates of depression across all cancer types, but the extent of these associations varied, highlighting the need for tailored psychological and financial support interventions for different groups of gynecological cancer patients.

### 3.3. Univariate and Multivariate Analysis of Factors Associated with Depression Symptoms in Middle-Aged and Young Female Cancer Patients

Table 2 delves into the factors potentially linked to the onset of depression symptoms among middle-aged and young female cancer patients, showcasing both univariate and multivariate analyses. Age emerged as a significant factor, with an increased likelihood of depression being associated with younger age. Economic stress was another significant predictor, with higher stress levels correlating with a higher risk of depression. Medical payment method also played a crucial role, as patients who paid out-of-pocket had a significantly higher risk of depression compared to those with insurance. Marital status showed that divorced and married participants had a reduced risk of depression compared to single participants. Ovarian cancer patients exhibited a lower risk of depression compared to those with cervical cancer. Other factors such as education, number of children, primary caregiver, work status, and cancer stage did not show significant associations with depression risk in the multivariate analysis. The presence of chronic disease was also a significant factor, with those having chronic diseases showing higher odds of depression. These findings underscore the complex interplay of demographic, socioeconomic, and clinical factors in the psychological well-being of cancer patients.

### 3.4. Performance of the Predictive Model for Depression among Gynecological Cancer Patients

**Figure 1:**
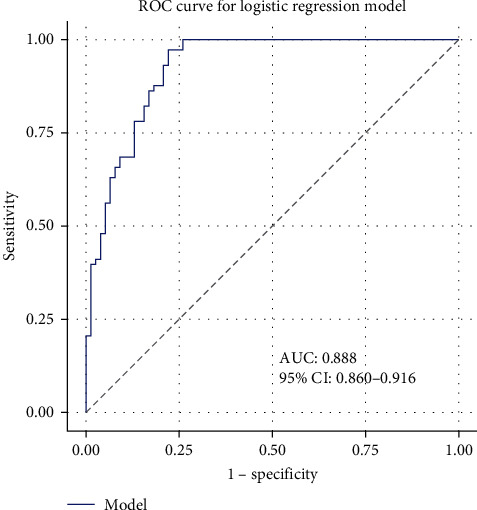
Receiver operating characteristic (ROC) curve for the predictive model of depression in middle-aged women with gynecological cancers. *Note*. This figure illustrates the performance of the logistic regression model developed to predict moderate to severe depressive symptoms among gynecological cancer patients in Southwest China. The area under the curve (AUC) is 0.888, indicating high discriminative ability of the model. The ROC curve plots sensitivity (true positive rate) against 1−specificity (false positive rate) at various threshold settings.

Our predictive model, designed to assess the risk of depression among gynecological cancer patients, demonstrates a high discriminative ability with an area under the curve (AUC) of 0.888 and a 95% confidence interval ranging from 0.860 to 0.916, indicating robust accuracy in distinguishing between patients who are likely to experience depression and those who are not. To ensure the robustness and generalizability of the predictive model, both internal and external validations were conducted. For internal validation, we performed 10-fold cross-validation on the original dataset, dividing the data into 10 subsets, with the model being trained on nine subsets and tested on the remaining subset in each iteration. The AUC for each fold was calculated, yielding an average AUC of 0.885 with a 95% confidence interval of 0.860–0.910 and a standard deviation of 0.012, demonstrating the model's stability and reliability. External validation was conducted using an independent dataset comprising 200 additional gynecological cancer patients, collected from a different time period and location within Southwest China. The model's performance on this dataset was evaluated, resulting in an AUC of 0.872 with a 95% confidence interval of 0.840–0.904 and a standard deviation of 0.016. These results highlight the model's consistent performance across different datasets and underscore its reliability and effectiveness in the early identification of depression risk in this patient population. The ROC curve illustrating the model's performance is shown in Figure 1.

## 4. Discussion

Our study provides a comprehensive analysis of the demographic and clinical characteristics of middle-aged and young female cancer patients, highlighting significant associations with depression symptoms. These findings contribute to the growing body of literature on the psychological well-being of cancer patients, particularly in the context of gynecological cancers.

The median age of 44 years in our cohort and the varied educational levels are consistent with previous studies on similar populations [12]. The fairly even distribution of marital status and work status categories indicates diverse social support and economic conditions faced by these patients, which are critical in cancer care and management. Previous research has emphasized the importance of social support in improving cancer patients' quality of life and psychological well-being [13]. The median number of children and the distribution of primary caregivers underscore the significant family and social responsibilities these patients bear, impacting their psychological well-being, as observed in other studies [14].

Our comparative analysis between patients with and without depression symptoms revealed significant differences, particularly highlighting the higher vulnerability of younger patients and those experiencing greater economic stress. This is in line with Ni et al. [15], who identified chronic pain and economic stress as major risk factors for depression in the Chinese middle-aged and elderly population. The association between chronic disease and depression observed in our study is consistent with findings from Hu et al. [16] and Wang et al.[12], highlighting the compounded impact of chronic conditions on mental health.

Economic stress emerged as a critical predictor of depression, with higher stress levels correlating with an increased risk of depression. This finding is supported by the systematic review by Smith et al. [17], which highlighted the financial burdens of cancer treatment as a significant factor contributing to psychological distress and nonadherence to treatment. The higher proportion of patients in the depression group paying out-of-pocket underscores the need for better financial support systems in cancer care. Peng et al. [18] also found that financial stress significantly impacts the psychological well-being of cancer patients, reinforcing the need for comprehensive financial support mechanisms.

The type of gynecological cancer and cancer stage also play roles in depression risk. Our stratified analysis demonstrated that ovarian cancer patients had a slightly younger median age, and vulvar cancer patients faced higher out-of-pocket expenses. These findings align with previous research indicating that different cancer types and stages can influence psychological outcomes and economic burdens [19]. This highlights the necessity for tailored interventions considering the specific type of gynecological cancer.

Our predictive model for assessing the risk of depression among gynecological cancer patients demonstrated high discriminative ability, with an AUC of 0.888. This robust performance underscores the model's reliability in identifying patients at risk for depression, which is crucial for early intervention. Fu et al. [20] highlighted the importance of psychosocial interventions in improving short-term survival rates in cancer patients, although the long-term benefits remain unclear. The consistent performance of our model across internal and external validations underscores its potential for widespread clinical application.

The significant associations between younger age, economic stress, and depression underscore the need for targeted psychosocial interventions. Martins-Klein et al. [21] emphasized the role of emotion regulation and resilience in mitigating psychological distress in cancer patients. Our findings suggest that interventions promoting resilience and coping strategies are essential for improving the psychological well-being of these patients. This is supported by studies demonstrating the positive effects of such interventions on quality of life and psychosocial adjustment [22, 23].

Our study found a high prevalence of depression (44%) among gynecological cancer patients in Southwest China. This prevalence is notably higher compared to previous studies conducted in different regions and populations. For instance, a study by Linden et al. [10] reported a prevalence of 22% among gynecological cancer patients in Canada, while a study in the United States by Carreira et al. [24] found a prevalence of 26% in a similar population. Several factors might contribute to the higher prevalence of depression observed in our study. The economic stress faced by patients in Southwest China may be more severe due to less developed healthcare infrastructure and higher out-of-pocket medical expenses. This financial burden can significantly impact the mental health of patients, exacerbating feelings of anxiety and depression. Additionally, cultural factors such as stigma associated with both cancer and mental health issues might lead to underreporting in other regions but more accurate reporting in our context. Furthermore, the younger median age of our cohort might also play a role, as younger patients often face more significant disruptions to their personal and professional lives, increasing their vulnerability to depression. In conclusion, the higher prevalence of depression in our study highlights the need for targeted psychological and financial support for gynecological cancer patients in Southwest China. Addressing these factors is crucial for improving the overall well-being and quality of life for these patients.

Despite these robust findings, our study has several limitations. Firstly, the cross-sectional design limits our ability to establish causality between the identified factors and depression symptoms. Longitudinal studies are needed to explore the causal relationships and long-term effects of these factors. Secondly, our study population is limited to a specific geographic region, which may limit the generalizability of the findings. Future research should include a more diverse population to validate our results. Lastly, self-reported measures of depression symptoms and economic stress may be subject to response bias, which could affect the accuracy of the findings.

In conclusion, our study highlights the complex interplay of demographic, socioeconomic, and clinical factors in the psychological well-being of middle-aged and young female cancer patients. The high prevalence of depression among younger patients and those facing economic stress calls for tailored psychological and financial support interventions. Future research should focus on longitudinal studies to explore the long-term effects of these interventions and further validate our predictive model in diverse populations. This approach is essential for improving the mental health outcomes of cancer patients and ensuring comprehensive care.

## Figures and Tables

**Table 1:** Comparative analysis of demographic and clinical characteristics between patients with and without depression symptoms.

Characteristics	Overall	No depression	Depression	*p* value
*n*	500	280	220	
Age, median (IQR)	44 (32, 56)	47 (33, 58)	41 (29, 52)	<0.001
Education (*n*; %)	—	—	—	0.388
Medium	152 (30.4%)	82 (29.3%)	70 (31.8%)	—
High	171 (34.2%)	103 (36.8%)	68 (30.9%)	—
Low	177 (35.4%)	95 (33.9%)	82 (37.3%)	—
Marital status (*n*; %)	—	—	—	0.207
Single	132 (26.4%)	64 (22.9%)	68 (30.9%)	—
Married	128 (25.6%)	74 (26.4%)	54 (24.5%)	—
Widowed	119 (23.8%)	68 (24.3%)	51 (23.2%)	—
Divorced	121 (24.2%)	74 (26.4%)	47 (21.4%)	—
Number children, median (IQR)	3 (1, 4)	3 (1, 4)	3 (1, 4)	0.609
Primary caregiver (*n*; %)	—	—	—	0.245
Spouse	105 (21%)	61 (21.8%)	44 (20%)	—
Other	108 (21.6%)	66 (23.6%)	42 (19.1%)	—
Children	101 (20.2%)	61 (21.8%)	40 (18.2%)	—
Friends	93 (18.6%)	45 (16.1%)	48 (21.8%)	—
Relatives	93 (18.6%)	47 (16.8%)	46 (20.9%)	—
Work status (*n*; %)	—	—	—	0.554
Retired	82 (16.4%)	45 (16.1%)	37 (16.8%)	—
Other	106 (21.2%)	67 (23.9%)	39 (17.7%)	—
Unemployed	105 (21%)	57 (20.4%)	48 (21.8%)	—
Full-time	107 (21.4%)	56 (20%)	51 (23.2%)	—
Part-time	100 (20%)	55 (19.6%)	45 (20.5%)	—
Medical payment method, (*n*; %)	—	—	—	<0.001
Insurance	156 (31.2%)	100 (35.7%)	56 (25.5%)	—
Out-of-pocket	194 (38.8%)	90 (32.1%)	104 (47.3%)	—
Government subsidy	74 (14.8%)	45 (16.1%)	29 (13.2%)	—
Other	76 (15.2%)	45 (16.1%)	31 (14.1%)	—
Economic stress, median (IQR)	3 (2, 5)	3 (2, 4)	4 (3, 5)	<0.001
Cancer diagnosis, (*n*; %)	—	—	—	0.203
Cervical	84 (16.8%)	39 (13.9%)	45 (20.5%)	—
Other	109 (21.8%)	62 (22.1%)	47 (21.4%)	—
Vulva	109 (21.8%)	59 (21.1%)	50 (22.7%)	—
Uterine	93 (18.6%)	53 (18.9%)	40 (18.2%)	—
Ovarian	105 (21%)	67 (23.9%)	38 (17.3%)	—
Cancer stage, (*n*; %)	—	—	—	0.434
III	113 (22.6%)	61 (21.8%)	52 (23.6%)	—
II	131 (26.2%)	69 (24.6%)	62 (28.2%)	—
IV	139 (27.8%)	77 (27.5%)	62 (28.2%)	—
I	117 (23.4%)	73 (26.1%)	44 (20%)	—
Chronic disease, (*n*; %)	—	—	—	<0.001
No	140 (28%)	90 (32.1%)	50 (22.7%)	—
Yes	360 (72%)	190 (67.9%)	170 (77.3%)	—

*Note*. IQR = Interquartile range. (*n;* %) = Number (percentage). Statistical significance was determined using appropriate tests: Mann–Whitney *U* test for continuous variables (age and economic stress), and *χ*^2^ test for categorical variables (education, marital status, primary caregiver, work status, medical payment method, cancer diagnosis, cancer stage, and chronic disease). Significant differences were observed in age, economic stress, medical payment method, and the presence of chronic disease between patients with and without depression symptoms, with *p* values indicating the level of significance.

**Table 2:** Univariate and multivariate analysis of factors associated with depression symptoms in middle-aged and young female cancer patients.

Characteristics	Total (*n*)	Univariate analysis		Multivariate analysis	
		Odds ratio (95% CI)	*p* value	Odds ratio (95% CI)	*p* value
Age	500	0.975 (0.962–0.987)	**<0.001**	0.976 (0.959–0.992)	**0.004**
Education	500	—	—	—	—
Medium	152	Reference	—	—	—
High	171	0.773 (0.497–1.203)	0.255	—	—
Low	177	1.011 (0.655–1.562)	0.960	—	—
Marital status	500	—	—	—	—
Single	132	Reference	—	Reference	—
Married	128	0.687 (0.421–1.120)	0.132	0.472 (0.244–0.913)	**0.026**
Widowed	119	0.706 (0.429–1.162)	0.171	0.562 (0.279–1.133)	0.107
Divorced	121	0.598 (0.363–0.986)	**0.044**	0.472 (0.238–0.936)	**0.032**
Number of children	500	0.973 (0.878–1.078)	0.601	—	—
Primary caregiver	500	—	—	—	—
Spouse	105	Reference	—	—	—
Other	108	0.882 (0.510–1.526)	0.654	—	—
Children	101	0.909 (0.521–1.585)	0.737	—	—
Friends	93	1.479 (0.843–2.594)	0.172	—	—
Relatives	93	1.357 (0.774–2.380)	0.287	—	—
Work status	500	—	—	—	—
Retired	82	Reference	—	—	—
Other	106	0.708 (0.393–1.274)	0.249	—	—
Unemployed	105	1.024 (0.573–1.830)	0.936	—	—
Full-time	107	1.108 (0.622–1.973)	0.728	—	—
Part-time	100	0.995 (0.553–1.789)	0.987	—	—
Medical payment method	500	—	—	—	—
Insurance	156	Reference	—	Reference	—
Out-of-pocket	194	2.010 (1.294–3.121)	**0.002**	2.115 (1.314–3.406)	**0.002**
Government subsidy	74	0.000 (0.000-Inf)	0.980	0.000 (0.000-Inf)	0.987
Other	76	0.000 (0.000-Inf)	0.980	0.000 (0.000-Inf)	0.986
Economic stress	500	1.636 (1.423–1.882)	**<0.001**	1.627 (1.350–1.962)	**<0.001**
Cancer diagnosis	500	—	—	—	—
Cervical	84	Reference	—	Reference	—
Other	109	0.657 (0.371–1.164)	0.150	0.521 (0.236–1.152)	0.107
Vulva	109	0.734 (0.415–1.300)	0.289	0.577 (0.266–1.249)	0.163
Uterine	93	0.654 (0.361–1.184)	0.161	0.543 (0.241–1.221)	0.140
Ovarian	105	0.492 (0.274–0.882)	**0.017**	0.393 (0.177–0.871)	**0.021**
Cancer stage	500	—	—	—	—
III	113	Reference	—	—	—
II	131	1.054 (0.636–1.746)	0.838	—	—
IV	139	0.945 (0.574–1.555)	0.823	—	—
I	117	0.707 (0.418–1.197)	0.197	—	—
Chronic disease	500	—	—	—	—
No	140	Reference	—	Reference	—
Yes	360	1.673 (1.189–2.356)	0.003	1.598 (1.126–2.270)	0.009

*Note*. Odds ratios (OR) with 95% confidence intervals (CI) are provided. The “Reference” category indicates the baseline group against which other groups were compared in the logistic regression analysis. *p* values are derived from logistic regression analysis. Values <0.05 are considered statistically significant. The bold font is intended to highlight the statistically significant *p*-values; specifically, values are considered statistically significant if the *p*-value is less than 0.05.

## Data Availability

The datasets analyzed during the current study are not publicly available due to privacy but are available from the corresponding author at a reasonable request.

## Supplementary Materials

Supplementary Materials Table S1: demographic and clinical characteristics of middle-aged and young female cancer patients (*N* = 500). This table presents the detailed demographic and clinical characteristics, including age, education, marital status, number of children, primary caregiver, work status, medical payment method, economic stress, cancer stage, and presence of chronic disease, for the overall cohort and subgroups based on cancer type (cervical, uterine, ovarian, vulva, and other). Table S2: comparative analysis of demographic and clinical characteristics between patients with and without depression symptoms for each type of gynecological cancer. This table provides a comparison of demographic and clinical characteristics between patients with and without depression symptoms across different types of gynecological cancers (cervical, uterine, ovarian, and vulvar cancer). It includes factors such as age, education, marital status, number of children, primary caregiver, work status, medical payment method, economic stress, cancer stage, and presence of chronic disease, along with their respective *p*-values indicating statistical significance.
