# Supplementary material for: Unveiling the Hidden Burden: Exploring the Psychological Impact of Gynecological Cancers and Predictive Modeling of Depression in Southwest China
Source: Depress Anxiety. 2024 Aug 2;2024:6512073. doi: 10.1155/2024/6512073 (PMC11918813; doi:10.1155/2024/6512073)
Supplement: Supplementary Materials — Table S1: demographic and clinical characteristics of middle-aged and young female cancer patients (N = 500). This table presents the detailed demographic and clinical characteristics, including age, education, marital status, number of children, primary caregiver, work status, medical payment method, economic stress, cancer stage, and presence of chronic disease, for the overall cohort and subgroups based on cancer type (cervical, uterine, ovarian, vulva, and other). Table S2: comparative analysis of demographic and clinical characteristics between patients with and without depression symptoms for each type of gynecological cancer. This table provides a comparison of demographic and clinical characteristics between patients with and without depression symptoms across different types of gynecological cancers (cervical, uterine, ovarian, and vulvar cancer). It includes factors such as age, education, marital status, number of children, primary caregiver, work status, medical payment method, economic stress, cancer stage, and presence of chronic disease, along with their respective p-values indicating statistical significance. [file 6512073.f1.docx]

**Supplementary Table 1: Demographic and Clinical Characteristics of Middle-aged and Young Female Cancer Patients (N=500)**

| **Characteristics** | **Overall (N=500)** | **Cervical (N=84)** | **Uterine (N=93)** | **Ovarian (N=105)** | **Vulva (N=109)** | **Other (N=109)** |
| --- | --- | --- | --- | --- | --- | --- |
| Age, median (IQR) | 44 (32, 56) | 45 (33, 57) | 46 (34, 58) | 43 (30, 55) | 44 (32, 56) | 42 (31, 54) |
| Education, n (%) |  |  |  |  |  |  |
| Medium | 152 (30.4%) | 25 (29.8%) | 28 (30.1%) | 32 (30.5%) | 33 (30.3%) | 34 (31.2%) |
| High | 171 (34.2%) | 28 (33.3%) | 32 (34.4%) | 37 (35.2%) | 38 (34.9%) | 36 (33.0%) |
| Low | 177 (35.4%) | 31 (36.9%) | 33 (35.5%) | 36 (34.3%) | 38 (34.9%) | 39 (35.8%) |
| Marital Status, n (%) |  |  |  |  |  |  |
| Single | 132 (26.4%) | 22 (26.2%) | 25 (26.9%) | 28 (26.7%) | 29 (26.6%) | 28 (25.7%) |
| Married | 128 (25.6%) | 21 (25.0%) | 24 (25.8%) | 27 (25.7%) | 28 (25.7%) | 28 (25.7%) |
| Widowed | 119 (23.8%) | 20 (23.8%) | 22 (23.7%) | 25 (23.8%) | 26 (23.9%) | 26 (23.9%) |
| Divorced | 121 (24.2%) | 21 (25.0%) | 22 (23.7%) | 25 (23.8%) | 26 (23.9%) | 27 (24.8%) |
| Number Children, median (IQR) | 3 (1, 4) | 3 (1, 4) | 3 (1, 4) | 3 (1, 4) | 3 (1, 4) | 3 (1, 4) |
| Primary Caregiver, n (%) |  |  |  |  |  |  |
| Spouse | 105 (21%) | 18 (21.4%) | 20 (21.5%) | 22 (21.0%) | 23 (21.1%) | 22 (20.2%) |
| Other | 108 (21.6%) | 19 (22.6%) | 21 (22.6%) | 23 (21.9%) | 24 (22.0%) | 22 (20.2%) |
| Children | 101 (20.2%) | 18 (21.4%) | 20 (21.5%) | 21 (20.0%) | 22 (20.2%) | 20 (18.3%) |
| Friends | 93 (18.6%) | 16 (19.0%) | 18 (19.4%) | 19 (18.1%) | 20 (18.3%) | 20 (18.3%) |
| Relatives | 93 (18.6%) | 16 (19.0%) | 18 (19.4%) | 20 (19.0%) | 20 (18.3%) | 19 (17.4%) |
| Work Status, n (%) |  |  |  |  |  |  |
| Retired | 82 (16.4%) | 14 (16.7%) | 15 (16.1%) | 17 (16.2%) | 17 (15.6%) | 19 (17.4%) |
| Other | 106 (21.2%) | 18 (21.4%) | 20 (21.5%) | 22 (21.0%) | 24 (22.0%) | 22 (20.2%) |
| Unemployed | 105 (21%) | 18 (21.4%) | 20 (21.5%) | 22 (21.0%) | 23 (21.1%) | 22 (20.2%) |
| Full-time | 107 (21.4%) | 18 (21.4%) | 20 (21.5%) | 23 (21.9%) | 23 (21.1%) | 23 (21.1%) |
| Part-time | 100 (20%) | 16 (19.0%) | 18 (19.4%) | 21 (20.0%) | 22 (20.2%) | 23 (21.1%) |
| Medical Payment Method, n (%) |  |  |  |  |  |  |
| Insurance | 156 (31.2%) | 28 (33.3%) | 30 (32.3%) | 33 (31.4%) | 34 (31.2%) | 31 (28.4%) |
| Out-of-pocket | 194 (38.8%) | 30 (35.7%) | 36 (38.7%) | 41 (39.0%) | 42 (38.5%) | 45 (41.3%) |
| Government subsidy | 74 (14.8%) | 12 (14.3%) | 14 (15.1%) | 15 (14.3%) | 16 (14.7%) | 17 (15.6%) |
| Other | 76 (15.2%) | 14 (16.7%) | 13 (14.0%) | 16 (15.2%) | 17 (15.6%) | 16 (14.7%) |
| Economic Stress, median (IQR) | 3 (2, 5) | 3 (2, 5) | 3 (2, 5) | 3 (2, 5) | 3 (2, 5) | 3 (2, 5) |
| Cancer Stage, n (%) |  |  |  |  |  |  |
| I | 117 (23.4%) | 21 (25.0%) | 24 (25.8%) | 27 (25.7%) | 28 (25.7%) | 27 (24.8%) |
| II | 131 (26.2%) | 23 (27.4%) | 27 (29.0%) | 28 (26.7%) | 30 (27.5%) | 23 (21.1%) |
| III | 113 (22.6%) | 20 (23.8%) | 22 (23.7%) | 24 (22.9%) | 26 (23.9%) | 21 (19.3%) |
| IV | 139 (27.8%) | 20 (23.8%) | 20 (21.5%) | 26 (24.8%) | 25 (22.9%) | 29 (26.6%) |
| Chronic Disease, n (%) |  |  |  |  |  |  |
| No | 140 (28%) | 24 (28.6%) | 26 (28.0%) | 29 (27.6%) | 30 (27.5%) | 31 (28.4%) |
| Yes | 360 (72%) | 60 (71.4%) | 67 (72.0%) | 76 (72.4%) | 79 (72.5%) | 78 (71.6%) |

Note: Data are presented as median (IQR) for continuous variables and number (percentage) for categorical variables. IQR: Interquartile Range. Economic Stress is rated on a scale from 1 to 5, where: 1: Very low economic stress; 2: Low economic stress; 3: Moderate economic stress; 4: High economic stress; 5: Very high economic stress.

**Supplementary Table 2: Comparative Analysis of Demographic and Clinical Characteristics Between Patients with and without Depression Symptoms for Each Type of Gynecological Cancer**

**Cervical Cancer**

| **Characteristics** | **NoDepression (n=39)** | **Depression (n=45)** | ***P* value** |
| --- | --- | --- | --- |
| Age, median (IQR) | 48 (34, 59) | 42 (32, 54) | 0.010 |
| Education, n (%) |  |  |  |
| Medium | 10 (25.6%) | 15 (33.3%) | 0.301 |
| High | 15 (38.5%) | 13 (28.9%) |  |
| Low | 14 (35.9%) | 17 (37.8%) |  |
| Marital Status, n (%) |  |  |  |
| Single | 10 (25.6%) | 12 (26.7%) | 0.701 |
| Married | 10 (25.6%) | 11 (24.4%) |  |
| Widowed | 10 (25.6%) | 10 (22.2%) |  |
| Divorced | 9 (23.1%) | 12 (26.7%) |  |
| Num Children, median (IQR) | 3 (1, 4) | 3 (1, 4) | 0.421 |
| Primary Caregiver, n (%) |  |  |  |
| Spouse | 8 (20.5%) | 10 (22.2%) | 0.601 |
| Other | 9 (23.1%) | 10 (22.2%) |  |
| Children | 8 (20.5%) | 10 (22.2%) |  |
| Friends | 7 (17.9%) | 9 (20.0%) |  |
| Relatives | 7 (17.9%) | 6 (13.3%) |  |
| Work Status, n (%) |  |  |  |
| Retired | 6 (15.4%) | 8 (17.8%) | 0.801 |
| Other | 9 (23.1%) | 9 (20.0%) |  |
| Unemployed | 8 (20.5%) | 10 (22.2%) |  |
| Full-time | 8 (20.5%) | 10 (22.2%) |  |
| Part-time | 8 (20.5%) | 8 (17.8%) |  |
| Medical Payment Method, n (%) |  |  | 0.041 |
| Insurance | 15 (38.5%) | 13 (28.9%) |  |
| Out-of-pocket | 10 (25.6%) | 15 (33.3%) |  |
| Government subsidy | 7 (17.9%) | 5 (11.1%) |  |
| Other | 7 (17.9%) | 12 (26.7%) |  |
| Economic Stress, median (IQR) | 3 (2, 5) | 4 (3, 5) | 0.032 |
| Cancer Stage, n (%) |  |  |  |
| I | 10 (25.6%) | 11 (24.4%) | 0.501 |
| II | 10 (25.6%) | 13 (28.9%) |  |
| III | 10 (25.6%) | 12 (26.7%) |  |
| IV | 9 (23.1%) | 9 (20.0%) |  |
| Chronic Disease, n (%) |  |  | 0.001 |
| No | 20 (51.3%) | 19 (42.2%) |  |
| Yes | 19 (48.7%) | 26 (57.8%) |  |

**Uterine Cancer**

| **Characteristics** | **NoDepression (n=53)** | **Depression (n=40)** | ***P* value** |
| --- | --- | --- | --- |
| Age, median (IQR) | 49 (35, 60) | 45 (33, 55) | 0.015 |
| Education, n (%) |  |  |  |
| Medium | 15 (28.3%) | 13 (32.5%) | 0.312 |
| High | 20 (37.7%) | 12 (30.0%) |  |
| Low | 18 (34.0%) | 15 (37.5%) |  |
| Marital Status, n (%) |  |  |  |
| Single | 15 (28.3%) | 10 (25.0%) | 0.721 |
| Married | 13 (24.5%) | 12 (30.0%) |  |
| Widowed | 13 (24.5%) | 10 (25.0%) |  |
| Divorced | 12 (22.7%) | 8 (20.0%) |  |
| Num Children, median (IQR) | 3 (1, 4) | 3 (1, 4) | 0.411 |
| Primary Caregiver, n (%) |  |  |  |
| Spouse | 11 (20.8%) | 9 (22.5%) | 0.641 |
| Other | 12 (22.6%) | 9 (22.5%) |  |
| Children | 11 (20.8%) | 8 (20.0%) |  |
| Friends | 10 (18.9%) | 8 (20.0%) |  |
| Relatives | 9 (17.0%) | 6 (15.0%) |  |
| Work Status, n (%) |  |  |  |
| Retired | 8 (15.1%) | 7 (17.5%) | 0.751 |
| Other | 12 (22.6%) | 8 (20.0%) |  |
| Unemployed | 10 (18.9%) | 8 (20.0%) |  |
| Full-time | 12 (22.6%) | 9 (22.5%) |  |
| Part-time | 11 (20.8%) | 8 (20.0%) |  |
| Medical Payment Method, n (%) |  |  | 0.032 |
| Insurance | 18 (34.0%) | 10 (25.0%) |  |
| Out-of-pocket | 12 (22.6%) | 16 (40.0%) |  |
| Government subsidy | 13 (24.5%) | 6 (15.0%) |  |
| Other | 10 (18.9%) | 8 (20.0%) |  |
| Economic Stress, median (IQR) | 3 (2, 5) | 4 (3, 5) | 0.025 |
| Cancer Stage, n (%) |  |  |  |
| I | 13 (24.5%) | 11 (27.5%) | 0.541 |
| II | 15 (28.3%) | 12 (30.0%) |  |
| III | 12 (22.6%) | 9 (22.5%) |  |
| IV | 13 (24.5%) | 8 (20.0%) |  |
| Chronic Disease, n (%) |  |  | 0.001 |
| No | 23 (43.4%) | 15 (37.5%) |  |
| Yes | 30 (56.6%) | 25 (62.5%) |  |

**Ovarian Cancer**

| **Characteristics** | **NoDepression (n=67)** | **Depression (n=38)** | ***P* value** |
| --- | --- | --- | --- |
| Age, median (IQR) | 47 (33, 57) | 43 (31, 54) | 0.011 |
| Education, n (%) |  |  |  |
| Medium | 18 (26.9%) | 14 (36.8%) | 0.301 |
| High | 26 (38.8%) | 11 (28.9%) |  |
| Low | 23 (34.3%) | 13 (34.2%) |  |
| Marital Status, n (%) |  |  |  |
| Single | 18 (26.9%) | 10 (26.3%) | 0.601 |
| Married | 16 (23.9%) | 11 (28.9%) |  |
| Widowed | 16 (23.9%) | 9 (23.7%) |  |
| Divorced | 17 (25.3%) | 8 (21.1%) |  |
| Num Children, median (IQR) | 3 (1, 4) | 3 (1, 4) | 0.511 |
| Primary Caregiver, n (%) |  |  |  |
| Spouse | 15 (22.4%) | 7 (18.4%) | 0.701 |
| Other | 16 (23.9%) | 8 (21.1%) |  |
| Children | 15 (22.4%) | 6 (15.8%) |  |
| Friends | 12 (17.9%) | 7 (18.4%) |  |
| Relatives | 12 (17.9%) | 10 (26.3%) |  |
| Work Status, n (%) |  |  |  |
| Retired | 11 (16.4%) | 6 (15.8%) | 0.801 |
| Other | 16 (23.9%) | 8 (21.1%) |  |
| Unemployed | 15 (22.4%) | 8 (21.1%) |  |
| Full-time | 14 (20.9%) | 10 (26.3%) |  |
| Part-time | 11 (16.4%) | 6 (15.8%) |  |
| Medical Payment Method, n (%) |  |  | 0.001 |
| Insurance | 21 (31.3%) | 12 (31.6%) |  |
| Out-of-pocket | 17 (25.4%) | 14 (36.8%) |  |
| Government subsidy | 18 (26.9%) | 7 (18.4%) |  |
| Other | 11 (16.4%) | 5 (13.2%) |  |
| Economic Stress, median (IQR) | 3 (2, 5) | 4 (3, 5) | 0.019 |
| Cancer Stage, n (%) |  |  |  |
| I | 16 (23.9%) | 11 (28.9%) | 0.421 |
| II | 18 (26.9%) | 10 (26.3%) |  |
| III | 17 (25.3%) | 7 (18.4%) |  |
| IV | 16 (23.9%) | 10 (26.3%) |  |
| Chronic Disease, n (%) |  |  | 0.001 |
| No | 34 (50.7%) | 15 (39.5%) |  |
| Yes | 33 (49.3%) | 23 (60.5%) |  |

**Vulvar Cancer**

| **Characteristics** | **NoDepression (n=59)** | **Depression (n=50)** | ***P* value** |
| --- | --- | --- | --- |
| Age, median (IQR) | 46 (34, 58) | 44 (32, 55) | 0.021 |
| Education, n (%) |  |  |  |
| Medium | 16 (27.1%) | 17 (34.0%) | 0.511 |
| High | 20 (33.9%) | 18 (36.0%) |  |
| Low | 23 (39.0%) | 15 (30.0%) |  |
| Marital Status, n (%) |  |  |  |
| Single | 17 (28.8%) | 12 (24.0%) | 0.601 |
| Married | 15 (25.4%) | 13 (26.0%) |  |
| Widowed | 15 (25.4%) | 11 (22.0%) |  |
| Divorced | 12 (20.3%) | 14 (28.0%) |  |
| Num Children, median (IQR) | 3 (1, 4) | 3 (1, 4) | 0.541 |
| Primary Caregiver, n (%) |  |  |  |
| Spouse | 14 (23.7%) | 9 (18.0%) | 0.701 |
| Other | 15 (25.4%) | 10 (20.0%) |  |
| Children | 13 (22.0%) | 10 (20.0%) |  |
| Friends | 11 (18.6%) | 9 (18.0%) |  |
| Relatives | 11 (18.6%) | 12 (24.0%) |  |
| Work Status, n (%) |  |  |  |
| Retired | 10 (16.9%) | 7 (14.0%) | 0.801 |
| Other | 14 (23.7%) | 10 (20.0%) |  |
| Unemployed | 13 (22.0%) | 10 (20.0%) |  |
| Full-time | 12 (20.3%) | 10 (20.0%) |  |
| Part-time | 10 (16.9%) | 8 (16.0%) |  |
| Medical Payment Method, n (%) |  |  | 0.021 |
| Insurance | 19 (32.2%) | 12 (24.0%) |  |
| Out-of-pocket | 16 (27.1%) | 16 (32.0%) |  |
| Government subsidy | 14 (23.7%) | 10 (20.0%) |  |
| Other | 10 (16.9%) | 12 (24.0%) |  |
| Economic Stress, median (IQR) | 3 (2, 5) | 4 (3, 5) | 0.029 |
| Cancer Stage, n (%) |  |  |  |
| I | 15 (25.4%) | 11 (22.0%) | 0.421 |
| II | 17 (28.8%) | 13 (26.0%) |  |
| III | 14 (23.7%) | 12 (24.0%) |  |
| IV | 13 (22.0%) | 14 (28.0%) |  |
| Chronic Disease, n (%) |  |  | 0.001 |
| No | 30 (50.8%) | 18 (36.0%) |  |
| Yes | 29 (49.2%) | 32 (64.0%) |  |
